# Effect of Clinic-Based and Asynchronous Video-Based Exercise on Clinic and Psychosocial Outcomes in Patients With Knee Osteoarthritis: Quasi-Experimental Study

**DOI:** 10.2196/58393

**Published:** 2025-03-26

**Authors:** Chidozie E Mbada, Henry Akintunji Awosika, Oluwatobi Ademola Sonuga, Micheal Akande, Tadesse Gebrye, Richard Woolf, Francis Fatoye

**Affiliations:** 1 Department of Health Professions Faculty of Health and Education Manchester Metropolitan University Manchester United Kingdom; 2 Department Of Medical Rehabilitation College Of Health Sciences Obafemi Awolowo University Ile-Ife Nigeria; 3 Federation of State Boards of Physical Therapy Alexandria, VA United States; 4 Lifestyle Diseases, Faculty of Health Sciences North-West University Potchefstroom South Africa

**Keywords:** knee osteoarthritis, video, physiotherapy, exercise, mobile phone, telehealth, telemedicine, randomized, controlled trial, asynchronous, rehabilitation, knees, joints, osteoarthritis, musculoskeletal, rheumatology, physical therapy

## Abstract

**Background:**

Telerehabilitation is promising for improving knee osteoarthritis, but the effect of different telerehabilitation strategies on knee osteoarthritis is unclear.

**Objective:**

This study aimed to examine the effect of a clinic-based strengthening exercise (CbSE) and asynchronous video-based strengthening exercise (AVbSE) on pain, range of motion, muscle strength, quality of life, and physical function among patients with knee osteoarthritis.

**Methods:**

A total of 52 consenting patients participated in this 8-week experimental study; they were assigned to the CbSE or AVbSE group at 2 different study sites. CbSE is a circuit exercise module comprising knee flexion and extension warm-up in sitting, quadriceps isometric setting, quadriceps strengthening exercise, hamstring clenches, wall squat, and a cooldown of knee flexion and extension. The AVbSE is an asynchronous video-based version of the CbSE.

**Results:**

This study spanned from March 31, 2021, to November 26, 2021. Eight out of 62 participants discontinued participation. Data collection and analysis have been completed. Significant differences were only observed in the mental health (*t*_50_=–3, *P*=.004), pain (*t*_39.4_*=*–3.6, *P*<.001), social support (*t*_50_*=*–2.7, *P*=.009), and social activities (*t*_50_*=*2.2, *P*=.03) domains of the Osteoarthritis Knee and Hip Quality of Life (OAKHQoL) questionnaire with higher scores in the AVbSE group at the end of week 4. At the end of week 8, significant differences were observed in mental health (*t*_50_=–2.1, *P*=.04) and pain (*t*_37.3_*=*–2.8, *P*=.008) measures with higher scores in AVbSE; however, a significantly higher score was observed in the CbSE group for the Quadruple Visual Analog Scale. No significant main effect of time was observed in this study, except in the muscle strength (*F*_2100_*=*1.5, *P=*.24), social support (*F*_2100_*=*2.5, *P*=.09), and social activity (*F*_2100_=0.7, *P*=.48) domains of the OAKHQoL questionnaire and activity limitation (*F*_2100_=0.1, *P*=.90), and performance restriction (*F*_2100_=1.3, *P*=.27) domains of the Ibadan Knee and Hip Osteoarthritis Outcome Measure (IKHOAM) questionnaire. There was no significant difference between groups in all OAKHQoL domains except social activities (mean 17.6, SD 1.2 vs 22.8, SD 1.2; *P*=.003) and average pain (2.8, SD 1.6 vs 2.3, SD 1.6; *P*=.03) with higher AVbSE mean scores. However, a higher score was observed for the CbSE group in the Quadruple Visual Analog Scale’s least pain domain (1.2, SD 0.2 vs 0.7, SD 0.2; *P*=.04). Also, interaction effects showed that AVbSE scores were significantly higher for the OAKHQoL questionnaire’s physical activity and mental health domains at all time points. However, the CbSE score was higher for the physical performance domain of the IKHOAM questionnaire in the eighth week.

**Conclusions:**

CbSE circuit training and its AVbSE variant effectively improve treatment outcomes and increase the quality of life of patients. While AVbSE was associated with higher improvement in most health-related quality of life domains, CbSE led to higher improvement in average pain.

**Trial Registration:**

Pan African Clinical Trial Registry PACTR202208515182119, https://pactr.samrc.ac.za/TrialDisplay.aspx?TrialID=23943

## Introduction

Osteoarthritis is a degenerative joint disease [1] affecting synovial joints and is a leading cause of pain and disability among adults [2]. Knee osteoarthritis is the most common type, with data indicating that 45% of people may develop symptomatic knee osteoarthritis in their lifetime [3]. Accordingly, knee osteoarthritis is the most prevalent type of osteoarthritis in Africans [4], accounting for 65%-78% of cases in hospitals in Nigeria [5]. Epidemiological studies have shown that obesity, microtrauma, knee joint alignment, repetitive use of joints, bone density, muscle weakness, and joint laxity are all local risk factors for knee osteoarthritis [6,7]. Further reports indicate that approximately 10% and 18% of men and women, respectively, present with myriad symptoms and radiological evidence, especially in about 30%-50% of adults aged 65 years and older [8,9]. Osteoarthritis of the knee is a prevalent musculoskeletal condition affecting older people, often accompanied by pain, physical disability, and reduced quality of life (QoL), with considerable economic burden on the health care system [10,11]. Research indicates that knee osteoarthritis occurs earlier in life, affecting younger adults more often than in previous years, likely due to increased obesity and joint injury [12].

The earlier occurrence and rising prevalence of knee osteoarthritis invite concern for effective management strategies to optimize the patient’s QoL [11] and the general well-being of the affected individuals [13]. Physiotherapy is a conservative approach that has been consistently recommended in the management of osteoarthritis [14]. The updated European League Against Rheumatism guidelines for the nonpharmacological core management of hip and knee osteoarthritis (recommendation 3) suggest that individuals with hip or knee osteoarthritis should be provided with an exercise program (such as strength, aerobic, flexibility, or neuromotor training) tailored to their physical function, preferences, and available services, with a progressive increase in intensity and level of difficulty [15]. The Osteoarthritis Research Society International guideline for the nonsurgical management of knee osteoarthritis also recommends land- and water-based exercises and muscle strengthening for pain management, improving physical function and QoL [16]. Accordingly, therapeutic exercise is the mainstay of physiotherapy for knee osteoarthritis as it is an effective and well-tolerated treatment [14]. Exercise intervention in knee osteoarthritis alleviates pain, improves physical performance, and optimizes social, domestic, occupational, and recreational participation [17]. Other potential benefits of exercise for this patient group include improvements in mobility and psychosocial health, as well as reduction of fall risk, body weight, and metabolic abnormalities [18]. Muscle strengthening is also a critical component of most exercise regimes for knee osteoarthritis [19]. Research shows that pain and disability can be reduced with aerobic walking and quadriceps strengthening in patients with knee osteoarthritis [20,21]. Self-management has also been increasingly shown to be an essential component of knee osteoarthritis management. Wu et al [22] found that self-management helped manage pain and stiffness and improve QoL in persons with knee osteoarthritis. Uritani et al [23] also found evidence for the benefits of self-management intervention in improving the self-efficacy of persons with knee osteoarthritis in handling pain and other symptoms. Accordingly, both the European League Against Rheumatism and Osteoarthritis Research Society International guidelines for managing knee osteoarthritis also recommend self-management in conjunction with exercise interventions for managing knee osteoarthritis [15,16].

Still, health care access–related issues can impede some patients with knee osteoarthritis from receiving exercise that is vital for their care [24]. Specifically, in medically underserved areas, physiotherapy services can be limited or lacking entirely [24,25]. These issues are particularly salient for individuals with low socioeconomic status, who also bear a greater burden of osteoarthritis [26]. These issues invite the need for innovative platforms that will help improve access to rehabilitative services and encourage self-management for patients with knee osteoarthritis [27].

Recently, telerehabilitation has been advocated to reduce the frequency of clinic visits, clinic waiting time, and costs incurred from transportation to the clinic, especially for patients living far distances from the clinic [24,28]. In addition, telerehabilitation has the potential to improve access to rehabilitation for patients in medically underserved countries like Nigeria and similar sub-Saharan African contexts. Medically underserved regions are characterized by a lack of adequate health care services, a high proportion of the population living in poverty, a high percentage of elderly residents, and a low ratio of health care providers to the population [29]. Africa is still facing a critical shortage of health workers despite dealing with the highest disease burden in the world [30,31]. Therefore, validated telerehabilitation platforms that may help overcome the challenges to accessing care faced by patients with knee osteoarthritis are necessary. Video-based telerehabilitation applications may effectively provide care in rural and resource-limited settings where internet-enabled digital services may be scarce or unavailable [24,25]. Particularly, asynchronous video-based telerehabilitation interventions can help overcome the time constraints of in-person and remote synchronous care [32]. Asynchronous digital health tools, also known as “store-and-forward” technology, allow for remote, non–real-time communication between providers and patients. This enables both parties to access the platform at their convenience [33]. These tools can address the limitations posed by poor or lack of internet access and coverage, which is common in Africa [34].

Given the prospect of asynchronous video-based telerehabilitation interventions in overcoming the barrier to health care access that persons with knee osteoarthritis may face, we propose an asynchronous video-based strengthening exercise program as a viable alternative. This study, therefore, seeks to compare the effect of a traditional clinic-based strengthening exercise (CbSE) program and an in-home asynchronous video-based strengthening exercise (AVbSE) program on clinical and psychosocial outcomes in patients with knee osteoarthritis. This study hypothesizes that the outcomes related to the in-home AVbSE would be comparable to CbSE over a period of 8 weeks.

## Methods

### Design

This study used a pretest-posttest quasi-experimental design.

### Participants

Eligible participants for the study included patients with radiologically confirmed knee osteoarthritis for not less than 3 months, who were 45 years and older, had a Kellgren-Lawrence grade of II and III, and were without any obvious deformities of the trunk or upper and lower extremities. Exclusion criteria were patients with other comorbidities such as acute inflammation, knee surgery, cardiovascular, respiratory, neurologic, and metabolic diseases, and pregnant women. Non–mobile phone owners after initial contact and screening were also excluded.

### Sample Size

Sample size estimation for the study was based on the equation c×π1(1–π1)+π2 (1–1π2)/(π1–π2)^2^ [35], where c=7.9 for 80% power, and π1 and π2 are proportion estimates (π1=0.25 and π2=0.65). Therefore, n=7.9 × (0.25 (1–0.25)+0.65 (1–0.65)/(0.25–0.65)=20.49, which is approximately 21. Hence, the calculated N is 42 (21 per group). To account for 10% possible attrition, the approximate minimum sample size was 46.

### Study’s Site and Sampling Procedure

This study was conducted at the Physiotherapy Departments of the Federal Medical Centre and Benue State University Teaching Hospital, both in Makurdi, Benue State, Nigeria. The study sites were selected purposefully based on the caseload at both centers and, second, one of the centers had fewer physiotherapists. The center with more physiotherapists was chosen as the control CbSE, while the other was the experimental AVbSE. Using 2 sites in this study was intended to minimize the spillover effect (spillover bias refers to an unintended effect that happens to those for whom the experimental intervention was not designed) by geographically separating the CbSE (control) from the AVbSE group. Also, the single-blind approach minimized the Hawthorne effect (that’s a situation where participants in the experimental or control group show exceptionally higher outcome scores or performance just because they are aware they are being studied). Thus, a research assistant who was not involved in the interventions conducted the recruitment and assessments. The study sample was randomly drawn from the list of patients with knee osteoarthritis attending both clinics.

### Outcomes

The primary outcomes in this study were pain, range of motion, and muscle strength, while osteoarthritis-specific QoL and physical function were the secondary outcomes. The instruments used in the study were the Quadruple Visual Analog Scale (QVAS), goniometer, tensiometer, and Ibadan Knee and Hip Osteoarthritis Outcome Measure (IKHOAM) questionnaire, and Osteoarthritis Knee and Hip Quality of Life (OAKHQoL) questionnaire.

***QVAS***

This tool was used to measure pain intensity based on current pain, average pain, least pain, and worst pain, respectively. The QVAS is a segmented numeric alternative to the visual analog scale where a whole number (0-10 integers) that best reflects the intensity of the pain for the different dimensions is chosen by the respondent [32]. The total score of the QVAS is derived by scoring 1+2+4, divided by 3, and multiplied by 10. Pain intensity can then be classified as low intensity (50) or high intensity (50) [36].

***Goniometer***

A goniometer (12-inch plastic [model 12-1000], Fabrication Enterprises) was used to measure the range of motion at the knee joint. It ranges from 0° to 180°.

***Tensiometer***

A tensiometer (model and pocket balance) was used to measure quadriceps muscle strength. The scale is from 0 kg to 100 kg.

***The IKHOAM Questionnaire***

The IKHOAM questionnaire was used to measure the activities of daily living and societal and physical performance. The IKHOAM questionnaire is a 3-domain, 33-item instrument consisting of an activity limitations domain comprising 25 activities of daily living that are performed by individuals with knee and hip osteoarthritis rated on a 5-point (0-4) ordinal scale, a participation restriction domain comprising 3 activities in societal participation rated on a 4 point (0-3) ordinal scale, and a physical performance tests domain including 5 tests rated by a clinician. These tests are (1) 250 m walk test rated on a 6-point (0-5) ordinal scale, (2) single-leg stance test rated on a 6-point (0-5) ordinal scale, (3) stair climbing test rated on a 5-point (0-4) ordinal scale, (4) squat test rated on a 5-point (0-4) ordinal scale, and (5) balance test rated on a 6-point (0-5) ordinal scale. The maximum obtainable score for the tests is 23 (5 scores for each of the 250 m walk test, 1 single-leg stance test, and one balance test, with 4 scores for the stair climbing test and squat test, respectively). The maximum obtainable score on the IKHOAM questionnaire is 232 (ie, 200+9+23). The score for each participant was calculated in percentage as a score divided by the total possible score and multiplied by 100. A low score on the IKHOAM questionnaire implies a low level of physical functioning ability, and a high score means a high level of physical functioning ability [37].

***The OAKHQoL Questionnaire***

The OAKHQoL questionnaire was used to assess the QoL. The OAKHQoL is the first specific QoL questionnaire for patients with knee and hip osteoarthritis. It is self-administered and comprises 43 items in 5 dimensions: physical activity, mental health, pain, social support, and social activities, plus 3 independent items. Dimension scores are standardized from 0 (worst QoL) to 100 (best QoL) [38].

### Procedure

The participants were recruited consecutively at each study site and followed through until they completed the 8-week treatment program. The participants were informed of the research’s purpose at the onset of the recruitment process. An informed written permission form, which was also translated into the local language by professionals, was given to every participant (N=64) who was evaluated for study eligibility. The number of people invited to participate (n=57), the number of people who rejected (n=3), the number of screened patients who were ineligible (n=4), and their justifications for declining participation were all tracked by a research assistant.

Each individual included in the study had a baseline assessment completed. Measurements included anthropometric variables such as height, weight, and BMI. Each participant’s information, including their age and gender, was recorded. All the questionnaires were applied, and the measurements of the range of motion of the knee joint and muscle strength (quadriceps) were taken at baseline, week 4, and week 8 of the study. Active graded exercises (active range of motion and quadriceps strengthening exercises) were administered to the patients in the 2 groups (CbSE and AVbSE) for 8 weeks.

The participants recruited into the AVbSE group received an asynchronous video containing exercises, instructions, and demonstrations of each exercise’s performance. The AVbSE was developed to be operable on a mobile device with at least an Android OS of 4.1 or an iPhone interface (iOS) for participants who own and could operate a smartphone. The video was also available as a CD drive for those who had or preferred it (n=8). The AVbSE video was an asynchronous video-based intervention intended for usage at home (a safe exercise space). The participants were encouraged to follow the video and complete the tasks thrice weekly. The exercises in the video include a warm-up of active seated knee flexion and extension, quadriceps isometric setting, quadriceps strengthening exercise, hamstring clenches, wall squats, and cool down of active seated knee flexion and extension. The participants in the AVbSE group were telemonitored with voice calls and SMSs to remind them of their exercise schedules and provide symptom reviews and feedback on performance. Also, the telemonitoring was aimed at guiding patients through the exercises and ensuring safety by asking patients to discontinue exercise in case of any adverse event. The CbSE involves the traditional in-person services exercise session led by a physiotherapist trained in the protocols in the AVbSE. The research assistant carried out all baseline treatment outcome assessments (at weeks 4 and 8) for both the CbSE and AVbSE at the clinics. The details of the exercises on the development and feasibility testing of a smartphone video-based exercise program for patients with knee osteoarthritis are already published in an earlier study by Mbada et al [24]. The exercise intervention was developed using a modified Delphi approach, which involved 3 rounds and included a panel of 4 experts and a patient with knee osteoarthritis. After reaching a consensus, the video program for knee osteoarthritis was developed, including 5 main types of exercises: seated knee flexion and extension, quadriceps isometric setting, quadriceps strengthening exercise, hamstring clenches, and wall squats [24]. The highlights of the intervention are (1) warm-up (active range of motion exercises) for 5-10 seconds, and the movement was performed up to 10 times; (2) quadriceps isometric setting repeated up to 10 times; (3) quadriceps strengthening exercise repeated up to 10 times; (4) hamstring clenches held for 10 seconds and then done 10 more times; (5) wall squat held for 10 seconds, then slide back up while keeping their backs firmly against the wall, and the movement was performed up to 10 times; and (6) cool down (active range of motion exercises) for 5-10 seconds, and the movement was performed up to 10 times [24].

### Statistical Analysis

Descriptive statistics of frequency, mean, and SD were used to summarize data, and an independent *t* test was used to compare patients’ general and clinical characteristics at baseline. A 2-way mixed-model ANOVA with repeated measures on the time points was used to determine the main effects of the two treatment programs (between-subject factor) and time points (within-subject factor) on the outcome parameters. Parametric tests were used in this study under the assumption that all the measurements are continuous variables on at least an interval scale, are normally distributed, and have equivalent variances. The α level was set at .05. The analysis was done using IBM SPSS (version 27).

### Ethical Considerations

This study was conducted according to the guidelines laid down in the Declaration of Helsinki and all procedures involving research study participants were approved by the Health Research Ethical Committee, Federal Medical Centre Makurdi, Nigeria (FMH/FMC/HRE/VL 1-24/03/21). Written informed consent was obtained from all participants.

## Results

**Figure 1:**
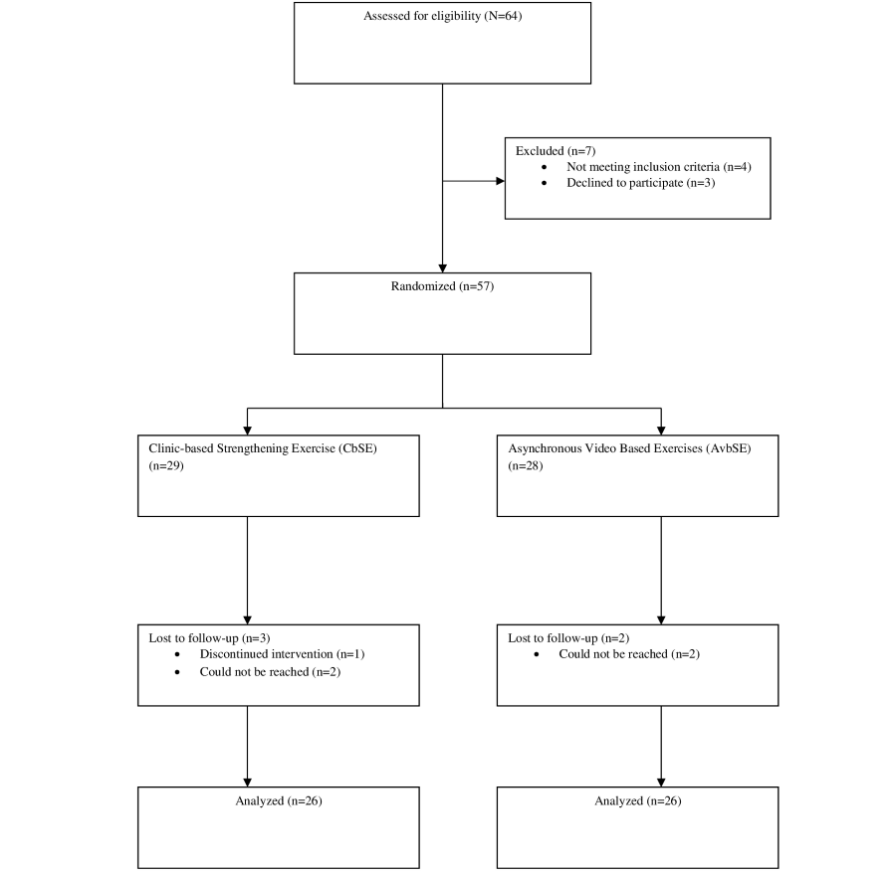
CONSORT diagram of the flow of participants through the study.

This study began on March 31, 2021, and ended on November 26, 2021. A total of 64 participants were recruited for the study but 8 participants discontinued. Data collection and analysis have been completed. The CONSORT (Consolidated Standards of Reporting Trials) flow diagram of the study is presented in Figure 1. Out of 64 patients, a total of 57 patients were found eligible for this study. There was a 12% loss to attrition based on the total number of participants recruited. Thus, 52 of 64 (81.3%) patients completed the study. The mean (SD) age, height, weight, and BMI of the participants were 56.2 (SD 7.5) years, 1.7 (SD 0.1) m, 68.8 (SD 7.6) kg, and 24.8 (SD 2.2) kg/m^2^, respectively. Participants in the two groups were comparable in their general characteristics (Table 1). Most participants in both groups were females (31/52, 59%). Civil servants, artisans, and businesspeople or traders comprised 15% (8/52), 21% (11/52), and 63% (33/52), of the participants, respectively. Also, a comparison of baseline measures in the selected outcomes is presented in Table 1. Both groups were comparable at baseline, except in OAKHQoL domains (*t*_39.9_=–4.1, *t*_42.5_=–4.7, *t*_38.3_=–5.8, *t*_50_=–4.2, and *t*_50_=6; *P*<.001), the physical performance domain of IKHOAM (*t*_50_=–2.2, *P=*.04), and the least pain domain of QVAS (*t*_50_=2.4, *P=*.02; Table 1).

Within-group effects (comparison of outcomes across baseline, fourth, and eighth week of the study) of CbSE and AVbSE are presented in Tables 2 and 3.

In Table 2, for a particular variable, mean values with different superscripts are significantly different (*P*<.05). When mean values have the same superscripts, they are not significantly different (*P*>.05; based on the least significant difference post hoc test).

**Table 1:** Comparison of the general characteristics and baseline measures between clinic-based strengthening exercise and asynchronous video-based telerehabilitation strengthening exercise groups (N=52).

Variables		CbSE^a^ (n=26)	AVbSE^b^ (n=26)	*t* test (*df*)	*P* value
Age (year), mean (SD)		56.2 (8.2)	56.2 (6.8)	–0.018 (50)	.16
Height (m), mean (SD)		1.7 (0.1)	1.7 (0.1)	–0.713 (50)	.81
Weight (kg), mean (SD)		67.3 (9.1)	70.3 (5.6)	–1.439 (50)	.08
BMI (kg/m^2^), mean (SD)		24.3 (2.5)	25.3 (1.7)	–1.640 (50)	.06
**Pain intensity, mean (SD)**					
	Average pain	3.2 (1.0)	3.8 (1.4)	–1.875 (44.4)	.07
	Least pain	1.8 (1.0)	1.2 (1.1)	2.385 (50)	.02
	Worst pain	6.8 (1.4)	7.1 (1.2)	–0.749 (50)	.46
	Total pain score	39.4 (9.5)	40.5 (9.7)	–0.432 (50)	.67
**OAKHQoL^c^ domain, mean (SD)**					
	Physical activities	36.8 (10.1)	53.1 (17.5)	–4.101 (39.9)	.001
	Mental health	25.3 (9.6)	41.9 (15.1)	–4.747 (42.3)	.001
	Pain	12.5 (2.5)	18.6 (4.7)	–5.791 (38.3)	.001
	Social support	24.1 (7.0)	31.3 (5.1)	–4.263 (50)	.001
	Social activities	23.5 (4.0)	17.2 (3.6)	5.994 (50)	.001
**IKHOAM^d^ domain** **, mean (SD)**					
	Activity limitation	142.2 (17.2)	144.2 (21.7)	–0.382 (50)	.70
	Participation restriction	6.0 (1.4)	6.5 (1.7)	–1.147 (50)	.26
	Physical performance	8.9 (3.1)	10.7 (2.8)	–2.167 (50)	.04

^a^CbSE: clinic-based strengthening exercise.

^b^AVbSE: asynchronous video-based telerehabilitation strengthening exercise.

^c^OAKHQoL: Osteoarthritis Knee and Hip Quality of Life.

^d^IKHOAM: Ibadan Knee and Hip Osteoarthritis Outcome Measure.

In Table 3, for a particular variable, mean values with different superscripts are significantly (*P*<.05) different. When mean values have the same superscripts, they are not significantly different (*P*>.05; based on the least significant difference post hoc test).

Results show significant differences in all the outcome parameters across the 3 time points of the study as shown in Tables 2 and 3. Also, a comparison of treatment outcomes between CbSE and AVbSE at weeks 4 and 8 of the study is presented in Table 4. The main effect of time was significant across all outcome parameters except muscle strength (*F*_2100_*=*1.5, *P=*.24), social support (*F*_2100_*=*2.5, *P*=.09), and social activities (*F*_2100_*=*0.7, *P=*.48) of the OAKHQoL questionnaire, as well as the activity limitation (*F*_2100_*=*0.1, *P*=.90) and performance restriction (*F*_2100_*=*1.3, *P=*.27) domains in the IKHOAM questionnaire as given in Table 5. Across all dimensions of the QVAS, the mean score reduced significantly from the fourth week to the eighth (*F*_2_=20.9-410.8, *P*<.001). Physical activities, mental health, and pain also showed a mean decrease across the time points. However, in the physical performance domain of IKHOAM, a significant increase in mean values was observed across the time points (Tables 2 and 3). Similarly, there were no significant differences in the treatment outcome between the two groups at the end of the 8 weeks of the study except for higher mean observed in mental health (17.5, SD 11.5 vs 11.6, SD 8.2; *t*_50_*=–*2.2, *P=.*04) and pain (23.6, SD 10.9 vs 5.5, SD 3.2; *t*_50_=–2.8, *P*=.008) in AVbSE group compared with CbSE group (Table 4).

A comparison of treatment outcomes between CbSE and AVbSE at weeks 4 and 8 of the study is presented in Table 4. The results of this study show that there were no significant differences in the treatment outcome between the 2 groups at the end of the fourth week of the study except for mental health (*t*_50_*=*–3.0, *P*=.004), pain (*t*_39.4_*=*–3.6, *P*<.001), social support (*t*_50_*=*–2.7, *P*=.009), and social activities (*t*_50_*=*2.2, *P*=.03) domains of the OAKHQoL questionnaire where the AVbSE group had significantly higher mean scores (Table 4). Similarly, there were no significant differences (*P*>.05) in the treatment outcome between the two groups at the end of the 8 weeks of the study except for a higher mean observed in mental health (17.5 vs 11.6; *t*_50_*=*–2.1, *P=*.04) and pain (23.6 vs 5.5; *t*_37.3_*=*–2.8, *P=*.008) in AVbSE group compared with CbSE group (Table 4). However, the CbSE group had a higher mean score in the current pain dimension of the QVAS (0.5 vs 0.1; *t*_41.4_*=*2.1, *P=.*04).

**Table 2:** Within-group comparison of treatment outcomes in the clinic-based strengthening exercise group (n=26).

Variables			Baseline		Fourth week		Eighth week			*F* test (*df*)		*P* value
Range of motion, mean (SD)			112.0 (7.3)^a^		108.5 (32.2)^a^		101.6 (44.3)^a^			497.7 (2)		.001
Muscle strength, mean (SD)			17.5 (4.5)^a^		19.2 (7.1)^a^		18.9 (8.9)^a^			260.4 (2)		.001
**Pain intensity** **, mean (SD)**												
	Current pain	1.8 (1.1)^a^		0.5 (0.9)^a^		0.5 (0.9)^a^			37.6 (2)		.001	
	Average pain	3.2 (1.0)^a^		2.3 (0.8)^a^		1.5 (0.9)^a^			289.5 (2)		.001	
	Least pain	1.8 (1.0)^a^		1.1 (1.1)^a^		0.7 (0.9)^a^			53.2 (2)		.001	
	Worst pain	6.8 (1.4)^a^		4.4 (1.8)^a^		2.6 (1.7)^a^			337.4 (2)		.001	
	Total pain score	39.4 (9.5)^a^		24.2 (9.8)^a^		15.1 (9.5)^a^			261.9 (2)		.001	
**OAKHQoL^b^ domain, mean (SD)**												
	Physical activities	36.8 (10.1)^a^		24.8 (12.9)^a^				17.8 (13.9)^a^	151.4 (2)		.001	
	Mental health	25.3 (9.6)^a^		16.0 (9.5)^a^				11.6 (8.2)^a^	106.9 (2)		.001	
	Pain	12.5 2.5)^a^		8.3 3.1)^a^		5.5 3.2)^a^			345.8 (2)		.001	
	Social support	24.1 7.0)^a^		25.1 8.6)^a^		23.6 10.9)^a^			354.2 (2)		.001	
	Social activities	23.5 4.0)^a^		22.7 7.6)^a^		22.2 10.5)^a^			312.4 (2)		.001	
**IKHOAM^c^ domain, mean (SD)**												
	Activity limitation	142.2 (17.2)^a^		143.7 45.2)^a^		145.3 (64.3)^a^			405.2 (2)		.001	
	Participation restriction	6.0 1.4)^a^		6.9 2.2)^a^				7.0 3.1)^a^	369.7 (2)		.001	
	Physical performance	8.9 3.1)^a^		11.2 4.4)^a^				13.0 6.4)^a^	188.1 (2)		.001	

^a^*P*<.05.

^b^OAKHQoL: Osteoarthritis Knee and Hip Quality of Life.

^c^IKHOAM: Ibadan Knee or Hip Osteoarthritis Outcome Measure.

**Table 3:** Within-group comparison of treatment outcomes in the asynchronous video-based strengthening exercise group (n=26).

Variables		Baseline	Fourth week	Eighth week	*F* test (*df*)	*P* value
Range of motion, mean (SD)		112.4 7.2)	112.1 (23.2)	95.3 47.5)	741.3 (2)	.001
Muscle strength, mean (SD)		17.4 4.6)	18.7 5.4)	15.8 8.7)	367.9 (2)	.001
**Pain intensity** **, mean (SD)**						
	Current pain	1.2 1.2)	0.5 0.9)	0.1 0.4)	20.9 (2)	.001
	Average pain	3.8 1.4)	2.8 1.1)	1.8 1.1)	231.8 (2)	.001
	Least pain	1.2 1.1)	0.8 1)	0.2 0.6)	25.6 (2)	.001
	Worst pain	7.1 1.2)	5.0 1.9)	3.3 1.9)	410.8 (2)	.001
	Total pain score	40.5 9.7)	27.9 10.9)	17.7 10.4)	335.7 (2)	.001
**OAKHQoL^a^, mean (SD)**						
	Physical activities	53.1 17.5)	30.9 17.0)	23.2 17.4)	146.8 (2)	.001
	Mental health	41.9 15.1)	25.4 12.8)	17.5 11.5)	143 (2)	.001
	Pain	18.6 4.7)	12.7 5.4)	9.3 6.3)	223.8 (2)	.001
	Social support	31.3 5.1)	31.4 7.9)	26.5 13.7)	482.1 (2)	.001
	Social activities	17.2 3.6)	18.6 5.7)	16.8 9.1)	285.2 (2)	.001
**IKHOAM^b^, mean (SD)**						
	Activity limitation	144.2 (21.7)	143.9 (35.5)	136 (67.7)	433.5 (2)	.001
	Participation restriction	6.5 1.7)	6.8 2.1)	6.4 3.3)	291.1 (2)	.001
	Physical performance	10.7 2.8)	11.3 3.3)	11.6 6.2)	253.2 (2)	.001

^a^OAKHQoL: Osteoarthritis Knee and Hip Quality of Life.

^b^IKHOAM: Ibadan Knee and Hip Osteoarthritis Outcome Measure.

The main effect of the group was found to be significant across all domains of the OAKHQoL measure with corrected *F* test values ranging from *F*_150_=21.9 (*P*<.001) on the pain domain to *F*_150_=6.4 (*P=*.01) on the physical activities domain*.* The main effect of the group was also significant for average pain intensity (*F*_150_=4.9, *P=*.03) and least pain intensity (*F*_150_=4.5, *P=*.04; Table 5)*.* Higher estimated marginal means scores were observed in the AvbSE treatment group across all domains of the OAKHQoL measure except the social activities domain, where the CbSE group was higher (22.8 vs 17.6; *F*_1_-score*=*9.9, *P=*.003). The average pain dimension of the QVAS score was higher in the AvbSE group (2.8 vs 2.3; *F*_1_-score=4.9, *P=*.03), while a higher mean was observed in the CbSE group for the least pain dimension (1.2 vs 0.7; *F*_1_-score=4.5, *P=*.04). The mean scores showing the difference between groups across all parameters are presented in Table 4.

**Table 4:** Comparison of treatment outcomes of clinic-based strengthening exercise and asynchronous video-based strengthening exercise at weeks 4 and 8 of the study (N=52).

		Week 4					Week 8			
Variables		CbSE^a^ (n=26)	AVbSE^b^ (n=26)	*t* test (*df*)	*P* value	CbSE (n=26)		AVbSE (n=26)	*t* test (*df*)	*P* value
Range of motion, mean (SD)		108.5 (32.2)	112.1 (23.2)	–0.459 (50)	.65	101.6 (44.3)		95.3 (47.5)	0.498 (50)	.62
Muscle strength, mean (SD)		19.2 (7.1)	18.7 (5.4)	0.265 (50)	.80	18.9 (9.0)		15.8 (8.7)	1.254 (50)	.22
**Pain intensity** **, mean (SD)**										
	Current pain	0.5 (0.9)	0.5 (0.9)	0.001 (50)	1.0	0.5 (0.7)		0.1 (0.4)	2.133 (41.4)	.04
	Average pain	2.3 (0.8)	2.8 (1.1)	–1.888 (50)	.07	1.5 (0.9)		1.9 (1.1)	–1.509 (50)	.14
	Least pain	1.1 (1.1)	0.8 (1.0)	0.953 (50)	.35	0.7 (1.0)		0.2 (0.6)	1.892 (40.9)	.06
	Worst pain	4.4 (1.8)	5.0 (1.9)	–1.192 (50)	.24	2.6 (1.7)		3.3 (2.0)	–1.363 (50)	.18
	Total pain score	24.3 (9.8)	27.9 (10.9)	–1.294 (50)	.20	15.1 (9.5)		17.7 (10.4)	–0.927 (50)	.36
**OAKHQoL^c^, mean (SD)**										
	Physical activities	24.8 (12.9)	30.9 (17)	–1.473 (46.6)	.15	17.8 (13.9)		23.2 (17.4)	–1.226 (50)	.23
	Mental health	16.0 (9.5)	25.4(12.8)	–3.000 (50)	.004	11.6 (8.2)		17.5 (11.5)	–2.139 (50)	.04
	Pain	8.3 (3.1)	12.7(5.4)	–3.644 (39.4)	.001	5.5 (3.2)		23.6 )	–2.782 (37.3)	.008
	Social support	25.1 (8.6)	31.4 (7.9)	–2.739 (50)	.009	23.6 (10.9)		26.5 (13.7)	–0.863 (50)	.39
	Social activities	22.7 (7.6)	18.6 (5.7)	2.196 (50)	.03	22.2 (10.5)		16.8 (9.1)	1.963 (50)	.06
**IKHOAM^d^, mean (SD)**										
	Activity limitation	143.7 (45.2)	144 (35.5)	–0.024 (50)	.98	145.3 (64.3)		136 (67.7)	0.506 (50)	.62
	Participation restriction	6.9 (2.2)	6.8 (2.1)	0.127 (50)	.90	7 (3.1)		6.4 (3.3)	0.646 (50)	.52
	Physical performance	11.2 (4.4)	11.3 (3.3)	–0.108 (50)	.92	13 (6.4)		11.6 (6.2)	0.837 (50)	.41

^a^CbSE: clinic-based strengthening exercise.

^b^AVbSE: asynchronous video-based strengthening exercise.

^c^OAKHQoL: Osteoarthritis Knee and Hip Quality of Life.

^d^IKHOAM: Ibadan Knee and Hip Osteoarthritis Outcome Measure.

**Table 5:** Main effects of treatment group, time points, and interaction effects.

Variables					*F* test (*df*)		*P* value		Partial^a^ η^2^			
**Range of motion**												
			Group		0.2 (1)		.90		0			
			Time		4.0 (2)		.02		0.074			
**Muscle strength**												
			Group		0.7 (1)		.41		0.013			
			Time		1.5 (2)		.24		0.028			
**Pain intensity**												
	**Current pain**											
			Group		2.1 (1)		.15		0.040			
			Time		42.4 (2)		.001		0.459			
	**Average pain**											
				Group		4.9 (1)		.03		0.089		
				Time		73.2 (2)		.001		0.594		
	**Least pain**											
			Group		4.5 (1)		.04		0.082			
			Time		31.4 (2)		.001		0.386			
	**Worst pain**											
			Group		2.2 (1)		.14		0.042			
			Time		117.3 (2)		.001		0.701			
	**Total pain score**											
			Group		1.2 (1)		.28		0.024			
			Time		143.4 (2)		.001		0.701			
**OAKHQoL^b^ domain**												
	**Physical activities**											
			Group		6.4 (1)		.014		0.114			
			Time		103.3 (2)		.001		0.674			
			Group×Time		6.1 (2)		.003		0.108			
	**Mental health**											
			Group		13.4 (1)		.001		0.211			
			Time		178.1 (2)		.001		0.781			
			Group×Time		14.2 (2)		.001		0.221			
	**Pain**											
			Group		21.9 (1)		.001		0.305			
			Time		99.0 (2)		.001		0.665			
	**Social support**											
			Group		8.6 (1)		.005		0.147			
			Time		2.5 (2)		.09		0.047			
	**Social activities**											
			Group		9.9 (1)		.003		0.166			
			Time		0.7 (2)		.48		0.015			
**IKHOAM^c^ domain**												
	**Activity limitation**											
			Group		0.1 (1)		.82		0.001			
			Time		0.1 (2)		.90		0.002			
	**Participation restriction**											
			Group		0.01 (1)		.92		0			
			Time		1.3 (2)		.27		0.026			
	**Physical performance**											
			Group		0.01 (1)		.90		0			
			Time		8.8 (2)		.001		0.150			
			Group×Time		3.6 (2)		.04		0.068			

^a^Partial η^2^: effect size.

^b^OAKHQoL: Osteoarthritis Knee and Hip Quality of Life.

^c^IKHOAM: Ibadan Knee and Hip Osteoarthritis Outcome Measure.

## Discussion

### Principal Findings

This study examined the effects of CbSE and AVbSE on clinical and psychosocial outcomes in patients with knee osteoarthritis. The average age of the patients was 56.2 (SD 7.5) years, and most were females. The gender pattern observed in this study might be a coincidence. However, more women are reported to seek care for their musculoskeletal conditions [8,39]. From this study, CbSE and AVbSE significantly improved clinical and psychosocial outcomes in patients with knee osteoarthritis.

### Comparison to Previous Work

These findings are consistent with previous reports that exercise interventions have beneficial clinical effects for patients with osteoarthritis in the lower limb [40,41]. The available evidence strongly supports the incorporation of exercise therapy as a fundamental component of nonpharmacological treatment for osteoarthritis of the knee [42]. Numerous studies have demonstrated that muscle strengthening and aerobic exercises can reduce pain and enhance physical function in individuals with mild to moderate knee osteoarthritis [43,44]. A systematic review by Turner et al [45] indicated that resistance training can improve pain and physical function, but the most effective resistance training regimen remains uncertain. Nevertheless, it appears that a total of 24 sessions over 8-12 weeks can yield significant benefits. A meta-analysis of 17 clinical trials involving 1705 patients revealed that resistance exercise contributes to a reduction in pain and stiffness, as well as an improvement in physical function for individuals with knee osteoarthritis [46]. Furthermore, it has been established that strength training exercises carried out over a period of 8 weeks, 3-5 times a week, can be both safe and effective for patients with knee osteoarthritis.

Furthermore, there is evidence for the effectiveness of resistance exercises conducted as a home exercise program for patients with knee osteoarthritis [40,41]. Doi et al [47] conducted a randomized controlled trial involving 142 patients with knee osteoarthritis. They found that a home-based quadriceps strengthening program involving 20 repetitions of a slow knee extension movement implemented 4 times daily for 8 weeks was as effective as nonsteroidal anti-inflammatory drugs for improved pain, stiffness, physical function, and QoL outcomes. However, in a systematic review, Saengpromma et al [48] found that a home exercise program with tracking improves pain and function and increases adherence and changes in behavior in older people compared with the untracked group. Doi et al [47] also corroborate that the effectiveness of home-based exercises in patients with knee osteoarthritis depends on patient adherence. Ettinger et al [49] and Thomas et al [50] submit that adherence to strengthening exercise is a key predictor of response in patients with knee osteoarthritis. However, the best way to deliver in-home strengthening exercises and promote adherence is still unclear.

Few studies emanating from West Africa explored digital rehabilitation for knee osteoarthritis. Odole and Ojo [28] implemented an in-home intervention of standardized exercise programs for patients with knee osteoarthritis, and adherence was monitored using telephone calls. The authors found that in-home telerehabilitation was comparable with clinic-based treatment in terms of improved QoL. Similarly, Ojoawo et al [51] compared clinic-based and telemonitored home-based interventions involving exercise pamphlets and telephone calls. They found that both interventions were effective in managing knee osteoarthritis, but the clinic-based intervention was better than the telemonitored home-based intervention. Both interventions implemented low-tech digital rehabilitation involving preintervention demonstration of exercises and the use of pictorial pamphlets as a guide. This is the first study to implement an asynchronous digital health tool for the rehabilitation of Nigerian patients with knee osteoarthritis. Implementing high-tech digital health interventions in the study setting is hamstrung by many factors, including poverty, lack of needed infrastructure (such as internet availability), and digital illiteracy [52,53].

In-home telerehabilitation creates opportunities to mitigate some of the challenges of home-based interventions (such as lack of support and good communication) using technological platforms [54]. Technology is not efficacious in itself; it depends on innovative ideas and providers’ creative abilities to ensure its use is sustainable [55]. According to Russell [56], telerehabilitation’s primary aim is to help patients receive equal access to rehabilitation services via images, sensors, virtual reality, and environments despite their impairments or barriers in accessibility. In physiotherapy, there has been an increase in the adoption of telecommunication technology, such as telephone or videoconferencing, to provide remote access to physiotherapy services when one-on-one contact is not feasible [28,57]. Telerehabilitation is a convenient way for patients to perform basic physiotherapy exercises on their own within their homes to avoid long-distance travel [58]. This study compared an asynchronous video-based telerehabilitation program with a clinic-based intervention. The treatment outcomes between the groups for both interventions are comparable except for average pain and least pain and OAKHQoL domains, where AVbSE produced significantly higher mean change in average pain and all domains of the OAKHQoL except social activities. Also, interaction effects show that the AVbSE had higher mean change across time points for PA and mental health OAKHQoL domain across all time points. Also, the CbSE score was only higher in the physical performance domain of the IKHOAM in the eighth week. Hence, AVbSE is a viable alternative to delivering physiotherapy for patients with knee osteoarthritis. The findings of this study support some related studies. Odole and Ojo [28] documented the efficacy of telephone-based rehabilitation on pain, physical function outcomes, and QoL in knee osteoarthritis. Chandra and Keerthi [58] also reported the efficacy of telerehabilitation using telephonic consultation and videoconferencing for patients with knee osteoarthritis, using exercise as a home-based treatment. Bennell et al [59] investigated the cost and clinical effectiveness of combining a physiotherapist-delivered intervention with a nurse-delivered telephone coaching for people with knee osteoarthritis. Better outcomes were achieved in the group of patients who received physiotherapy treatment and the nurse-delivered telephone coaching than those who received only physiotherapy treatment. According to Adhikari et al [60], telephone-based treatments used in telerehabilitation were both feasible and efficient, significantly reducing pain caused by various musculoskeletal problems in resource-limited settings. Another study by Multani et al [61] found signiﬁcant improvement in pain, muscle strength, and functional ability within 4 weeks of telerehabilitation in patients with knee osteoarthritis.

This study supports other studies, including reviewed literature that suggest that muscle strengthening interventions improve pain and physical functioning in persons with mild to moderate osteoarthritis of the knee. Also, the study reveals that asynchronous video-based exercises effectively manage patients with knee osteoarthritis and produce comparable effects in physiologic and psychosocial outcomes to conventional clinic-based physiotherapy. Furthermore, the findings of this study confirm an earlier submission by Winters [62] that telerehabilitation brings the hope of enabling access for all, as well as helping address societal challenges in the delivery of rehabilitative services once barriers such as distance and reimbursement are overcome. Also, according to Lamplot et al [63], as technology improves in remote and rural locations, initial knee examination and follow-up visits may also be conducted virtually [63]. The findings of this study support earlier advocacy on the necessity for the development and implementation of telerehabilitation services to support persons with potentially disabling conditions who may have limited access to rehabilitation services [64,65].

### Clinical Implications

The study’s relevance lies in its provision of evidence on digital rehabilitation to expand access to rehabilitation for individuals with knee osteoarthritis in Nigeria and similar medically underserved contexts. The exercises used in this study were developed based on available evidence and expert opinions following a Delphi study. The interventions were applied in accordance with evidence from a systematic review, which showed that exercise programs lasting 8-12 weeks, with 3-5 sessions per week, are safe and effective for patients with knee osteoarthritis. This study aligns with recent recommendations for self-management in patients with chronic conditions [66], particularly for those with osteoarthritis [67]. Digital interventions have been beneficial in promoting self-management among patients with various conditions [68,69], including osteoarthritis [70,71]. Using asynchronous video-based exercises as digital rehabilitation for patients with knee osteoarthritis would improve access to physiotherapy services, eliminate geographical and distance barriers, and reduce the burden of clinic visits and waiting times.

### Study Limitations

This study has some potential limitations that may affect the generalizability of findings. As such, some characteristics of the sample must be considered. First, the significant difference in the baseline parameter between the intervention and control group regarding the OAKHQoL domains, physical performance domain of IKHOAM, and least pain domain of QVAS may be a source of bias on the outcome of the interventions. Second, most participants were primarily middle-aged adults who were largely uneducated; this may affect the ease of use of technological tools. Third, this study used a dual clinical approach to recruiting participants, which could have potential inherent selection bias. Selecting multiple sites for this study may introduce selection bias related to patient demographics or treatment practices, which could independently influence the outcomes of the intervention. However, the findings of the study suggest that the general characteristics of patients at baseline were comparable across groups, indicating that the potential for selection bias is limited. Although both clinics are classified as tertiary health care institutions in Nigeria, the distinctive attributes of teaching hospitals often make them the preferred option for patients seeking specialized and comprehensive health care. As such, the control site, which is the teaching hospital in this case, may have more standardized treatment practices, potentially leading to performance bias and affecting treatment outcomes. Fourth, it is challenging to guarantee the adherence of the participants in the AVbSE group, as it is commonly reported in asynchronous tele-exercise programs, despite the telemonitoring used in this study. Also, asynchronous tele-exercises offer scheduling flexibility to patients but do not provide for real-time interface and response from the provider.

Furthermore, excluding patients without mobile phones from data collection may be a limitation of this study. While mobile phone ownership is relatively high worldwide, it is still a problem in specific populations, such as adults in rural or developing areas, and children [72]. In Nigeria, smartphone penetration is increasing and is expected to reach around 60% by 2025 [73]. However, mobile phone ownership in poorer areas is still limited, with only 58% of Nigerians in urban areas and 32% in rural areas owning smartphones in 2022 [74]. Therefore, the pervasive digital divide or digital poverty in many African settings hinders the continent from fully benefiting from digital technology [75]. For instance, the lack of mobile phone ownership could impede the implementation of mobile health interventions in Africa [76]. Researchers conducting self-report research using mobile phones should be aware that providing a mobile phone to standardize participant response platforms can impact response behavior [72].

### Direction for Future Work

Future research investigating the short- and long-term effects of telerehabilitation among a larger population of patients with knee osteoarthritis from multiple sites is recommended to validate the findings of this study.

### Conclusion

Clinic-based strengthening exercise circuit training and its asynchronous video-based variant have been found to effectively reduce pain, impairment, and disability and increase patients’ QoL. The video-based variant was associated with higher improvement in most health-related QoL domains. In comparison, clinic-based strengthening exercise circuit training led to higher improvement in pain intensity.

## Abbreviations

AvbSE: asynchronous video-based strengthening exercises
CbSE: clinic-based strengthening exercises
CONSORT: Consolidated Standards of Reporting Trials
IKHOAM: Ibadan Knee and Hip Osteoarthritis Outcome Measure
OAKHQoL: Osteoarthritis Knee and Hip Quality of Life
QoL: quality of life
QVAS: Quadruple Visual Analog Scale

## Data Availability

The datasets generated during and analyzed during this study are available from the corresponding author.
